# Socioeconomic Disparities in Functional Status in a National Sample of Patients With Rheumatoid Arthritis

**DOI:** 10.1001/jamanetworkopen.2021.19400

**Published:** 2021-08-04

**Authors:** Zara Izadi, Jing Li, Michael Evans, Nevin Hammam, Patricia Katz, Alexis Ogdie, Lisa G. Suter, Jinoos Yazdany, Gabriela Schmajuk

**Affiliations:** 1 Department of Epidemiology and Biostatistics, University of California, San Francisco; 2 Division of Rheumatology, School of Medicine, University of California, San Francisco; 3 Departments of Medicine and Epidemiology, Perelman School of Medicine, University of Pennsylvania, Philadelphia; 4 Yale University, New Haven, Connecticut; 5 VA Medical Center, West Haven, Connecticut; 6 VA Medical Center, San Francisco, California

## Abstract

**Importance:**

Little is known about the association of poverty with functional status (FS) in patients with rheumatoid arthritis (RA) who use rheumatology care.

**Objectives:**

To examine the association between socioeconomic status (SES) and FS among patients with RA and to evaluate the association between SES and functional declines over time in patients who received at least some rheumatology care.

**Design, Setting, and Participants:**

This cohort study used data from the American College of Rheumatology’s Rheumatology Informatics System for Effectiveness (RISE) registry between January 1, 2016, and December 31, 2018. Analyses included all adult patients with a confirmed RA diagnosis (ie, had ≥2 encounters associated with RA *International Classification of Diseases *codes ≥30 days apart) and at least 1 FS score documented between 2016 and 2018 seen at participating rheumatology practices. Data analysis was conducted from April to December 2020.

**Exposures:**

The Area Deprivation Index (ADI), a zip code–based indicator of neighborhood poverty, was used as a proxy for SES. ADI scores were categorized into quintiles.

**Main Outcomes and Measures:**

FS measures included Multidimensional Health Assessment Questionnaire (MDHAQ), Health Assessment Questionnaire Disability index, and Health Assessment Questionnaire–II. Cross-sectionally, mean FS scores were compared across ADI quintiles. Longitudinally, among patients with at least 2 FS scores, multilevel multivariate regression computed the probability of functional decline, defined as a change greater than the minimum clinically important difference, across ADI quintiles. In a subgroup analysis, whether disease activity mediated the association between SES and functional decline was examined.

**Results:**

Of the 83 965 patients included in the study, 66 649 (77%) were women, and 60 037 (72%) were non-Hispanic White. Mean (SD) age was 63.4 (13.7) years. MDHAQ was the most reported FS measure (56 928 patients [67.8%]). For all measures, mean (SD) FS score was worse at lower SES levels (eg, for MDHAQ quintile 1: 1.79 [1.87]; quintile 5: 2.43 [2.17]). In longitudinal analyses, the probability of functional decline was 14.1% (95% CI, 12.5%-15.7%) in the highest SES quintile and 18.9% (95% CI, 17.1%-20.7%) in the lowest SES quintile. The association between SES and functional decline was partially mediated (7%; 95% CI, 4%-22%) by disease activity.

**Conclusions and Relevance:**

In this cohort study of patients with RA, worse FS and faster declines in functioning over time were observed in patients with lower SES. These findings provide a framework for monitoring disparities in RA and for generating evidence to spur action toward achieving health equity.

## Key Points

**Question:**

Are there socioeconomic disparities in functional status among individuals with rheumatoid arthritis seen in rheumatology practices?

**Findings:**

In this national cohort of 83 965 individuals with rheumatoid arthritis, functional status was statistically significantly worse across each successively lower quintile of socioeconomic status (SES). In addition, the probability of functional decline over the study period was statistically significantly higher in individuals with low SES (18.9% in the lowest SES quintile) compared with individuals with high SES (14.1% in the highest SES quintile).

**Meaning:**

These findings suggest that disparate health outcomes exist among individuals with rheumatoid arthritis seen in rheumatology practices.

## Teaser

This cohort study examines the association between socioeconomic status and functional status among patients with rheumatoid arthritis and evaluates the association between socioeconomic status and functional declines over time in patients who received at least some rheumatology care.

## Introduction

Social determinants of health can profoundly affect health outcomes, including patients with rheumatic disease. Although rheumatoid arthritis (RA) is the most common autoimmune rheumatic disease and a leading cause of disability, disparities in clinical and patient-reported outcomes in this condition are poorly characterized. In the last 2 decades, the treatment of RA has seen unprecedented advances, and the promise of a life without significant disability is possible for many patients. However, it remains unknown whether treatment advances have benefited all populations. Research to monitor disparities and guide policies to reduce them is therefore relevant and timely.

To date, most studies of social determinants of health among patients with RA conducted have come from single centers.^1,2,3,4^ These studies have examined important sociodemographic factors, such as poverty, but only in limited samples, which may not represent the national picture. Single-center studies may also have limited power to examine patient-reported outcomes, such as disease activity or functional status (FS). The preservation of FS is one of the most important issues in the long-term care for patients with RA,^5^ as it is closely associated with joint damage, quality of life, employment, and disability in this population.^6^ FS assessment using validated tools is a key part of high-quality rheumatology care and a nationally endorsed RA-specific performance measure that has been operationalized as an electronic health record (EHR)–enabled measure. This provides a unique opportunity to examine FS on a national level.

Although studies have shown that poor access to rheumatology care leads to worse RA disease outcomes,^7^ less is known about whether disparities exist among populations receiving at least some rheumatology care. In this study, we used the American College of Rheumatology’s Rheumatology Informatics System for Effectiveness (RISE), a national, EHR-enabled registry that passively collects data on all patients seen by participating practices,^8^ to examine the association between socioeconomic status (SES) and FS among individuals with RA across the United States and to evaluate the association between SES and functional decline over time.

## Methods

### Data Source, Time Lines, and Study Population

Data were derived from the RISE registry. As of December 2018, RISE held validated data from 1113 clinicians in 226 practices, representing approximately 32% of the US clinical rheumatology workforce. Patients included in this study were 18 years or older, had at least 2 visits with RA *International Classification of Diseases, Ninth Revision *and *Tenth Revision *codes^9^ (including 714.x, M05x, and M06x; excluding M06.4) at least 30 days apart, and had at least 1 FS measure documented between January 1, 2016, and December 31, 2018. The Western institutional review board and the University of California, San Francisco, Committee on Human Research approved this study. A waiver of informed consent was granted by the institutional review board because the research presented no more than minimal risk to participants; could not practicably be done without the waiver; could not practicably be done without identifiable information; will not adversely affect rights and welfare of participants with the waiver; and will provide participants with additional pertinent information after participation, whenever it is appropriate. We followed the Strengthening the Reporting of Observational Studies in Epidemiology (STROBE) reporting guidelines for cohort studies.

### SES

We used the Area Deprivation Index (ADI) as a proxy for patient’s SES. The ADI has been examined in various studies for its association with health outcomes.^10^ It is a 9-digit, zip code–based indicator of neighborhood socioeconomic disadvantage, which incorporates neighborhood income, education, employment, and housing quality domains (range 1-100; higher score indicates lower SES).^11^ The ADI scores were categorized into quintiles, with the first ADI quintile representing the highest SES level, and the fifth ADI quintile representing the lowest SES level.

### FS Measures

The FS measures used in this study included Health Assessment Questionnaire Disability index (HAQ),^12^ Health Assessment Questionnaire–II (HAQ-II),^13^ and Multidimensional Health Assessment Questionnaire (MDHAQ)^14^ and were collected during the course of routine clinical care. A total of 109 practices had patients with at least 1 of these measures documented in RISE. The HAQ includes 20 items related to activities of daily living over the past week.^12^ HAQ-II^13^ and MDHAQ,^15^ both with 10 items, are intended to be short replacements for the HAQ. For all 3 measures, higher scores represent more disability. Practices using 1 to 30–point or 0 to 3–point scales for MDHAQ were all converted to 0 to 10–point scales, the most commonly used MDHAQ scale, by dividing the scores by 10 or multiplying by 3.3333, respectively. Practices using 1 to 24–point or 0 to 10–point scales for HAQ and HAQ-II were all converted to a 0 to 3–point scale, the most commonly used scale, by dividing by 8 or 3.3333, respectively. If a patient had multiple FS measure scores recorded during the study period, we only included 1 of them according to the following hierarchy: MDHAQ, HAQ-II, HAQ.

### Covariates

Other patient characteristics included sex, race/ethnicity (non-Hispanic White, Hispanic, African American, Asian, other [ie, American Indian or Alaska Native and Native Hawaiian or other Pacific Islander] or multiracial/mixed), age, smoking status (ever or never), and insurance type (private, Medicare, any Medicaid, or other). Race/ethnicity data were collected from EHRs because race/ethnicity is tied to other unmeasured social determinants of health. Local workflows to collect this information vary and can either rely on patient self-report or, in some cases, assignment of categories by staff. Clinical characteristics included number of rheumatology visits during the study period and disease activity as assessed by the Clinical Disease Activity Index (CDAI),^16^ which incorporates tender and swollen joint counts as well as a patient’s and physician’s global assessment of RA disease activity. Additionally, information on medications used during the study period was extracted and categorized into 4 categories (biologic disease-modifying antirheumatic drugs [bDMARDs], targeted synthetic DMARDs [tsDMARDs], conventional systemic DMARDs [csDMARDs], and glucocorticoids [GCs]), allowing each individual to be assigned to more than 1 medication category. Practice characteristics included practice size (number of clinicians); practice type (single-specialty group practice, multispecialty group practice, solo practitioner, or health system); practice location according to the 9 US geographic divisions; and EHR vendor (NextGen, eClinicalWorks, Amazing Charts, GE Centricity, or other).

### Statistical Analysis

Descriptive statistics were calculated for sociodemographic, clinical, and practice characteristics. To identify any notable trends, the distribution of sociodemographic factors, including age, sex, and race/ethnicity, were compared across ADI quintiles. Two sets of analyses were conducted to assess the association between SES and FS scores. First, in a cross-sectional analysis using only the most recent FS measure reported during the study period, we assessed the association between SES and FS. Mean scores for each FS measure across ADI quintiles were reported. Statistical significance of a linear trend across the quintiles of ADI was tested separately for each FS measure using a Wald test on marginal linear predictions. A 2-tailed *P* < .05 was considered statistically significant.

Second, we performed longitudinal analyses to further investigate the association between SES and functional decline over time. Patients with at least 2 FS scores, measured at least 12 months apart, were included in this analysis. Change in FS score was defined as most recent score minus the next most recent score (baseline FS score) that was at least 12 months prior. Functional decline (yes or no) was based on minimum clinically important difference for each FS measure and defined as increases in scores greater than 1.2 for MDHAQ, greater than 0.25 for HAQ, and greater than 0.28 for HAQ-II.^17^ We used pooled multilevel logistic regression to examine the association between ADI quintiles as the exposure and functional decline over the study period as the outcome, across the 3 measures, after adjusting for potential confounders, including age, sex, race/ethnicity, baseline FS score (categorized as quartiles for consistency across the measures), medications used, number of visits during the study period, time elapsed between the 2 FS scores, and accounting for within-practice correlations. The first ADI quintile was used as the reference category. Computed probabilities of functional decline were reported for ADI quintiles and other covariates included in the models.

Finally, we evaluated whether the association between SES and functional decline was mediated by disease activity among a subgroup of individuals for whom CDAI scores were available during the study period. For each individual, we extracted the most recent CDAI documented within 6 months prior to the baseline FS score. Given that the relative change over time in FS scores using these instruments is small, the mediation analysis^18^ was implemented using a binary variable for ADI (greater or less than the median score) to facilitate detection of differences in the probability of functional decline between ADI groups. No formal power analysis was done, as all eligible patients in RISE were included in the study. All data analyses were conducted in Stata version 16.0 (StataCorp).

## Results

A total of 83 965 patients with RA from 109 practices were included in the cross-sectional analysis. Most were women (66 649 [77%]) and non-Hispanic White (60 037 [72%]), with a mean (SD) age of 63.4 (13.7) years (Table 1). Patients had a median (interquartile range [IQR]) ADI score of 43 (23-66). The first and fifth ADI quintiles corresponded to ADI scores of 1 to 18 and 72 to 100, respectively. The number of patients with RA varied by practice, with a median (IQR) of 1505 (835-2892). Nearly 80% of patients (66 062 [79%]) were from single-specialty group practices. A total of 21 650 patients (26%) had a documented CDAI score within 6 months of the most recent FS assessment. Among these patients, 3519 (16%) were in remission and 7514 (35%), 7261 (34%), and 2256 (16%) had low, moderate, and high disease activity,^19^ respectively. While characteristics of patients included in the analyses were generally comparable with those of patients who were excluded from the study, there were some notable differences; patients who were excluded were predominantly from practices with solo practitioners, had lower disease activity, and received fewer DMARDs.

**Table 1.** Characteristics of the Cross-Sectional and Longitudinal Cohorts and Rheumatoid Arthritis Patients Excluded From Analyses

Characteristic	Patients, No. (%)		
	Cross-sectional cohort (n = 83 965)	Longitudinal cohort (n = 35 385)	Patients excluded^a^ (n = 105 510)
Age, mean (SD), y	63.4 (13.7)	63.7 (13.1)	63.1 (13.9)
Sex			
Male	19 316 (23.0)	7991 (22.6)	24 477 (23.2)
Female	64 649 (77.0)	27 394 (77.4)	81 033 (76.8)
Race/ethnicity			
Non-Hispanic White	60 037 (71.5)	26 120 (73.8)	67 440 (63.9)
Hispanic	4304 (5.1)	1444 (4.1)	9361 (8.9)
African American	6900 (8.2)	2876 (8.1)	7981 (7.6)
Asian	1106 (1.3)	495 (1.4)	1338 (1.3)
Other or multiracial^b^	4996 (6.0)	1802 (5.1)	5265 (5.0)
Unknown	6622 (7.9)	2648 (7.5)	14 125 (13.4)
Insurance			
Private	22 584 (26.9)	10 891 (30.8)	38 136 (36.1)
Medicare	24 698 (29.4)	12 521 (35.4)	35 658 (33.8)
Any Medicaid	2051 (2.4)	888 (2.5)	3189 (3.0)
Other	1763 (2.1)	701 (2.0)	6556 (6.2)
Unknown	32 869 (39.2)	10 383 (29.3)	21 971 (20.8)
Area Deprivation Index, median (IQR)^c^	43 (23-66)	43 (22-67)	45 (25-67)
Practice type			
Single-specialty group practice	66 062 (78.7)	28 049 (79.3)	71 734 (68.0)
Multispecialty group practice	12 062 (14.4)	5683 (16.1)	14 367 (13.6)
Solo practitioner	5636 (6.7)	1587 (4.5)	17 432 (16.5)
Health system	205 (0.2)	66 (0.2)	1977 (1.9)
Clinicians per practice, median (IQR), No.	6 (4-10)	7 (5-10)	5 (2-8)
Eligible patients per practice, median (IQR), No.	1505 (835-2892)	1732 (966-3067)	1472 (930-2555)
Geographic division			
New England	953 (1.1)	203 (0.6)	1789 (1.7)
Mid-Atlantic	9068 (10.8)	2888 (8.2)	12 863 (12.2)
East North Central	10 486 (12.5)	5121 (14.5)	17 623 (16.7)
West North Central	7972 (9.5)	3361 (9.5)	8734 (8.3)
South Atlantic	32 715 (39.0)	12 060 (34.1)	24 845 (23.6)
East South Central	11 688 (13.9)	6638 (18.8)	7551 (7.2)
West South Central	2843 (3.4)	1378 (3.9)	16 164 (15.3)
Mountain	3719 (4.4)	2353 (6.7)	5418 (5.1)
Pacific	4521 (5.4)	1383 (3.9)	10 523 (10.0)
EHR vendor			
NextGen	62 950 (75.0)	27 279 (77.1)	30 867 (29.3)
eClinicalWorks	9349 (11.1)	3608 (10.2)	30 130 (28.6)
Amazing Charts	1396 (1.7)	373 (1.1)	2567 (2.4)
GE Centricity	3600 (4.3)	2236 (6.3)	2758 (2.6)
Other	6670 (8.0)	1889 (5.3)	39 188 (37.1)
Visits per patient during the study period, median (IQR), No.	8 (5-12)	10 (7-13)	8 (5-11)
Medications prescribed during the study period^d^			
bDMARDs	35 387 (42.1)	17 651 (49.9)	23 327 (22.1)
tsDMARDs	5992 (7.1)	2995 (8.5)	2695 (2.6)
csDMARDs	56 632 (67.5)	25 267 (71.4)	31 568 (29.9)
GCs	54 339 (64.7)	24 323 (68.7)	44 427 (42.1)
Clinical Disease Activity Index^e^			
Total patients with measure, No.	21 650	2053	15 160
Remission	3519 (16.3)	350 (17.1)	4223 (27.9)
Low	7514 (34.7)	766 (37.3)	4966 (32.8)
Moderate	7261 (33.5)	635 (30.9)	3932 (25.9)
High	3356 (15.5)	302 (14.7)	2029 (13.4)

Abbreviations: bDMARDs, biologic disease-modifying antirheumatic drugs; csDMARDS, conventional systemic disease-modifying antirheumatic drugs; EHR, electronic health record; GCs, glucocorticoids; IQR, interquartile range; tsDMARDs, targeted synthetic disease-modifying antirheumatic drugs.

^a^ Patients with a diagnosis of rheumatoid arthritis were excluded from analyses if there was no documentation of functional status during the study period.

^b^ Other races included American Indian or Alaska Native and Native Hawaiian and other Pacific Islander.

^c^ The Area Deprivation Index has a range of 1 to 100, with higher scores indicating lower socioeconomic status.

^d^ Medication categories are not mutually exclusive. bDMARDs include abatacept, adalimumab, anakinra, belimumab, canakinumab, certolizumab, denosumab, eculizumab, etanercept, golimumab, infliximab, natalizumab, rituximab, secukinumab, siltuximab, tocilizumab, sarilumab, ustekinumab, and vedolizumab; csDMARDs, mercaptopurine, azathioprine, cyclophosphamide, cyclosporine, leflunomide, methotrexate, mycophenolate mofetil, mycophenolic acid, sulfasalazine, tacrolimus, chloroquine, and hydroxychloroquine; GCs, prednisone, dexamethasone, hydrocortisone, methylprednisolone, cortisone, prednisolone, triamcinolone, and betamethasone; and tsDMARDs, apremilast, tofacitinib, baricitinib, and upadacitinib.

^e^ Scores obtained within 6 months before the most recent functional status score in the cross-sectional cohort; within 6 months before the baseline functional status score in longitudinal cohort; and the earliest documented Clinical Disease Activity Index within the study period for the patients excluded.

Age and sex distributions were comparable across ADI quintiles in both the cross-sectional and longitudinal cohorts. The racial/ethnic distribution varied significantly across SES levels in both cohorts, with higher proportions of non-Hispanic White and Asian patients in the first ADI quintile compared with the fifth quintile and more African American and Hispanic patients in the fifth ADI quintile compared with the first quintile (eTable in the Supplement).

**Figure 1.**
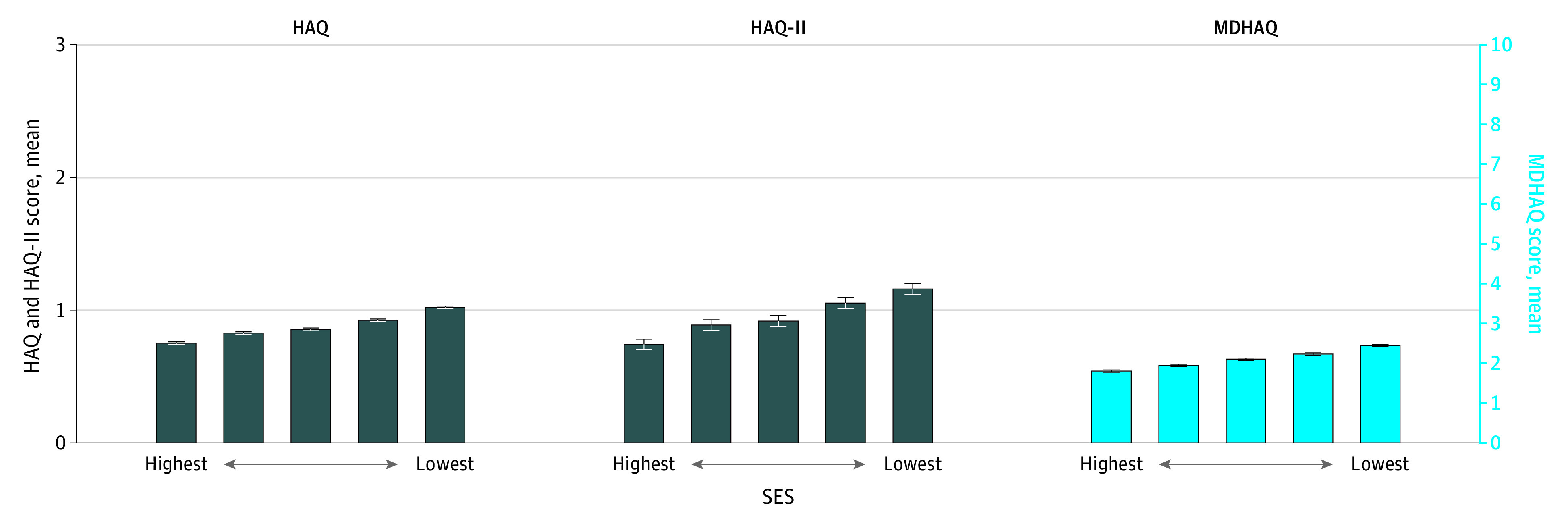
Mean Functional Status Measure Scores Across Quintiles of Area Deprivation Index in the Cross-Sectional Analysis Error bars represent 95% confidence interval for the means. Statistical significance of a linear trend across the quintiles of socioeconomic status (SES) was tested separately for each functional status measure using a Wald test on marginal linear predictions; all tests were statistically significant at the *P* < .05 level. HAQ indicates Health Assessment Questionnaire Disability index; HAQ-II, Health Assessment Questionnaire–II; MDHAQ, Multidimensional Health Assessment Questionnaire.

MDHAQ was the most commonly reported FS measure (56 928 patients [68%]) in the cross-sectional cohort, followed by HAQ (20 488 [24%]) and HAQ-II (6549 [8%]). A similar distribution was observed in the longitudinal cohort. In the cross-sectional analysis, mean (SD) scores of MDHAQ (range 0-10), HAQ (range 0-3), and HAQ-II (range 0-3) were 2.1 (2.0), 0.9 (0.6), and 1.0 (0.7), respectively. The mean (SD) FS score was higher, indicating greater disability, at higher ADI quintiles across all 3 measures (eg, for MDHAQ quintile 1: 1.79 [1.87]; quintile 5: 2.43 [2.17]) (Figure 1).

**Figure 2.**
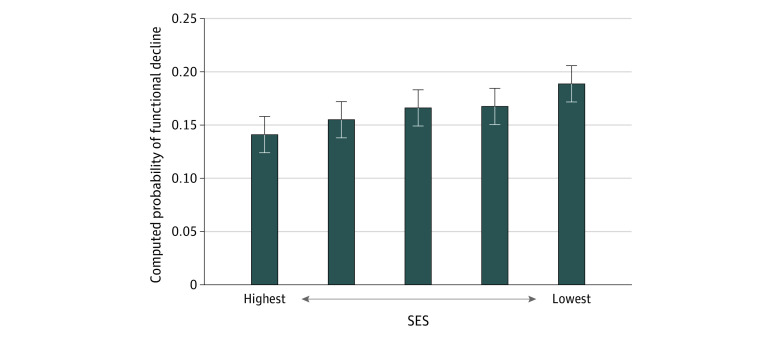
Computed Probabilities of Functional Decline Across Quintiles of Area Deprivation Index in the Longitudinal Analysis The multivariate model was adjusted for age, sex, race/ethnicity, baseline functional status, medication prescribed, number of visits, duration between the 2 functional status scores, and within-practice correlations. Error bars represent 95% CIs for computed probabilities. Statistical significance of a linear trend across the quintiles of socioeconomic status (SES) was tested using a Wald test on marginal linear predictions; the test was statistically significant at the *P* < .05 level.

A total of 35 385 patients with RA were included in the pooled adjusted longitudinal analysis. The probability of functional decline was higher at higher ADI quintiles (Figure 2). Computed probabilities of functional decline and corresponding 95% CIs are reported in Table 2; 18.9% (95% CI, 17.1%-20.7%) of patients worsened in the fifth ADI quintile while 14.1% (95% CI, 12.5%-15.7%) of patients worsened in the first ADI quintile. Consistent results were obtained after additionally adjusting for smoking status or practice characteristics (data not shown).

**Table 2.** Computed Probabilities of Functional Decline Among Individuals With Rheumatoid Arthritis During the Study Period^a^

Characteristic	Probability (95% CI), %	
	Unadjusted probabilities	Adjusted probabilities^b^
Age^c^		
25th percentile (56 y)	15.9 (14.5-17.3)	16.0 (14.5-17.4)
Median (65 y)	16.1 (14.7-17.5)	16.4 (14.9-17.8)
75th percentile (73 y)	16.3 (14.9-17.7)	16.7 (15.2-18.2)
Sex^c^		
Male	16.4 (15.0-17.8)	16.6 (15.2-18.1)
Female	15.1 (13.5-16.6)	15.3 (13.7-16.8)
Race/ethnicity^c^		
Non-Hispanic White	15.7 (14.2-17.1)	16.0 (14.5-17.5)
Hispanic	18.5 (16.0-21.0)	18.5 (16.0-21.0)
African American	18.4 (16.4-20.4)	18.0 (16.0-20.0)
Asian	15.8 (12.3-19.3)	16.5 (12.8-20.1)
Other or multiracial^d^	15.4 (13.2-17.6)	16.2 (14.0-18.5)
Unknown or declined to answer	16.6 (14.6-18.7)	16.5 (14.5-18.6)
ADI quintile^c^		
1 (highest SES level)	14.3 (12.7-15.9)	14.1 (12.5-15.7)
2	15.2 (13.7-16.8)	15.5 (13.9-17.1)
3	16.3 (14.7-17.9)	16.6 (14.9-18.3)
4	16.2 (14.6-17.8)	16.8 (15.1-18.4)
5 (lowest SES level)	18.4 (16.7-20.1)	18.9 (17.1-20.7)
Visits, No.^c^		
25th percentile (7)	15.6 (14.2-17.0)	16.0 (14.5-17.5)
Median (10)	15.9 (14.5-17.3)	16.2 (14.7-17.7)
75th percentile (13)	16.3 (14.8-17.7)	16.4 (15.0-17.9)
Medications during study period^e^		
bDMARDs	17.3 (15.8-18.8)^c^	17.4 (15.8-18.9)^c^
tsDMARDs	18.6 (16.6-20.6)^c^	19.6 (17.5-21.7)^c^
csDMARDs	16.1 (14.6-17.5)	16.4 (14.9-17.9)
GCs	17.8 (16.4-19.4)^c^	18.2 (16.6-19.7)^c^

Abbreviations: ADI, area deprivation index; bDMARDs, biologic disease-modifying antirheumatic drugs; csDMARDs, conventional systemic disease-modifying antirheumatic drugs; GCs, glucocorticoids; SES, socioeconomic status; tsDMARDs, targeted synthetic disease-modifying antirheumatic drugs.

^a^ Functional decline (yes or no) was based on minimum clinically important difference for each measure, defined as increases in scores of greater than 1.2 for the Multidimensional Health Assessment Questionnaire, greater than 0.25 for the Health Assessment Questionnaire Disability index, and greater than 0.28 for the Health Assessment Questionnaire–II.

^b^ Models additionally adjusted for time elapsed between the 2 functional status scores and baseline functional status score.

^c^ *P* < .05 in global Wald test.

^d^ Other races included American Indian or Alaska Native and Native Hawaiian and other Pacific Islander.

^e^ bDMARDs include abatacept, adalimumab, anakinra, belimumab, canakinumab, certolizumab, denosumab, eculizumab, etanercept, golimumab, infliximab, natalizumab, rituximab, secukinumab, siltuximab, tocilizumab, sarilumab, ustekinumab, and vedolizumab; csDMARDs, mercaptopurine, azathioprine, cyclophosphamide, cyclosporine, leflunomide, methotrexate, mycophenolate mofetil, mycophenolic acid, sulfasalazine, tacrolimus, chloroquine, and hydroxychloroquine; GCs, prednisone, dexamethasone, hydrocortisone, methylprednisolone, cortisone, prednisolone, triamcinolone, and betamethasone; and tsDMARDs, apremilast, tofacitinib, baricitinib, and upadacitinib.

The mediation analysis included 2053 patients with at least 1 CDAI score within 6 months prior to the baseline FS score. On average, RA disease activity mediated a small but statistically significant proportion (7%; 95% CI, 4%-22%) of the association between SES and functional decline in this longitudinal cohort (Table 3).

**Table 3.** Subgroup Mediation Analysis^a^

Association between ADI and functional decline^b^	Estimate (95% CI)^c^
Total association	0.042 (0.013-0.071)
Average mediation through CDAI	0.003 (0.001-0.006)
Average direct association	0.039 (0.009-0.069)
Total association mediated through CDAI, %	0.073 (0.042-0.222)

Abbreviations: ADI, area deprivation index; CDAI, clinical disease activity index.

^a^ In a subgroup of patients with rheumatoid arthritis with at least 2 functional status scores during the study period at least 12 months apart and a documented CDAI score within 6 months before the first functional status score.

^b^ Functional decline (yes or no) was based on minimum clinically important difference for each measure, defined as increases in scores of greater than 1.2 for the Multidimensional Health Assessment Questionnaire, greater than 0.25 for the Health Assessment Questionnaire Disability index, and greater than 0.28 for the Health Assessment Questionnaire–II.

^c^ Estimates represent percentage-point increases in the probability of functional decline during the study period associated with an ADI score greater than the median compared with an ADI score lower than the median. Mediator and outcome models adjusted for age, sex, race/ethnicity, and baseline functional status score.

## Discussion

Although rheumatoid arthritis is a prevalent autoimmune disease and a leading cause of disability among US adults, health disparities in RA outcomes are not well characterized. In this study, we performed what we believe is the largest national evaluation of FS outcomes in individuals with RA. Among patients seen by US rheumatologists, we found significant disparities in FS, with worse FS across each successive lower quintile of SES. In addition, FS was more likely to decline over time among patients in lower SES groups. These findings persisted even when controlling for the number of visits that patients had to a rheumatology practice, suggesting that utilization of rheumatology care is not sufficient to eliminate disparate health outcomes for individuals with RA.

While recent advances in the management of RA, such as the expansion of biologic therapies over the past 2 decades, have led to dramatic improvements in health outcomes, it is not clear whether these gains have been shared equally across patient sociodemographic groups. A few prior studies^1,3^ have shown that some RA outcomes remain uneven across the population, with individuals from racial/ethnic minority groups experiencing higher disease activity and more disability compared with White patients. National data sources such as RISE provide an important opportunity to systematically monitor and generate evidence to spur action toward achieving health equity.

Our findings are consistent with published reports of disparities in patient-reported outcomes among patients with rheumatic disease. In people with self-reported arthritis, lower levels of household income and higher levels of community poverty were associated with poor mental health outcomes (as measured by the Medical Outcomes Study Short Form 12, version 2, mental component summary or the Center for Epidemiological Studies Depression [CES-D] scale),^20^ higher levels of functional impairment and disability (as measured by the Medical Outcomes Study Short Form 12. version 2, physical component summary or HAQ), and worse health-related quality of life (as measured by the Centers for Disease Control and Prevention Health-Related Quality of Life).^21^

Research in other musculoskeletal conditions, including a community-based cohort of patients with osteoarthritis, has found that lower individual-level and community-level SES, lower educational attainment, and nonmanagerial occupations were associated with worse function, pain, and stiffness and more disability.^22,23^ Studies of social determinants of health have been more extensive in patients with systemic lupus erythematosus (SLE). Poverty has been shown to be associated with physical and mental health outcomes (Systemic Lupus Activity Questionnaire, a self-reported assessment of SLE symptoms; the Medical Outcomes Study Short Form–36, physical functioning score; and CES-D) and to play a critical role in the accumulation of damage (Brief Index of Lupus Damage) in patients with SLE.^24,25^ In a recent qualitative study, patients with SLE reported that poverty necessitated prioritizing personal resources to deal with food, medical care, and housing insecurity on a daily basis and to relegate their management of SLE to occurrences of disease flares. Study participants also reported that exposure to crime in their neighborhoods was a stressor that could trigger flares of disease activity.^26^ Low income has also been shown to be associated with fewer rheumatology visits in a large cohort of patients with SLE,^27^ suggesting that while patients with lower incomes may have access to health care through public insurance programs, the presence of health insurance alone does not ensure equal utilization of care.

Research on social determinants is limited in RA. It is unclear why disparities in RA have not been highlighted, particularly in relation to patient-reported outcomes. Nonetheless available data suggest significant health disparities in RA; lower disease activity (Disease Activity Score in 28 joints) and better function (HAQ) was reported among White patients compared with patients from other racial/ethnic groups with RA patients at a university hospital in California.^1^ The same pattern was observed for disease activity by language (English compared with languages other than English) and immigrant status (US-born compared with immigrant) at the same clinic.^1^

Access to care is often thought to be a major driver of disparities, yet our findings reveal disparities even among RA patients who used specialist care, and they persisted over time. Our mediation analysis suggests that improving disparities in FS will require understanding the reasons for higher disease activity among patients in the lowest SES groups. Hypotheses include that these disparities may result from events that preceded access to specialist care, such as delays in initial diagnosis and treatment, leading to a more severe disease course, or from either disparate treatment or factors that impede adequate treatment, such as depression, low health literacy, and lower adherence to treatments.^28^ Medication adherence can be impacted by costs^29,30,31,32,33,34^ or patients’ trust in their clinicians and/or the health care system.

Programs that directly attempt to reduce disparities among low-income populations are needed and should rely on systems that measure, track, and aim to improve disparate outcomes. Such programs might include a variety of interventions that have been successful in other chronic diseases, such as chronic disease management programs^35,36,37,38,39,40,41,42,43,44,45^ and programs that build partnerships between health systems and community-based organizations, such as fresh food markets, smoking cessation classes, and free support groups.^46^ Targeted outreach^47^ that is culturally and linguistically tailored to patients with low SES might be another strategy to help preserve FS among individuals with RA. It is important to acknowledge that these approaches are not sufficient to address social determinants of health, such as educational and employment equity, residential segregation, or poverty, which are also fundamental drivers of persistent health disparities. The greatest health impacts will come from interventions that address SES factors that drive health disparities across multiple domains.

### Strengths and Limitations

This study has important strengths; to our knowledge, it is the first to provide a national view of socioeconomic disparities in FS among patients with RA. Our study population was broader than prior studies that have been limited to single sites, with findings that are generalizable across the United States among practices participating in RISE. The study was also sufficiently powered to detect a significant association between SES and functional decline despite small changes in FS over time. Additionally, this study includes clinical information from rheumatologists, implying higher fidelity in RA diagnoses and FS assessments, compared with administrative data^9^ or survey-based methods.^48^

The study also has limitations. The study population may not fully represent patients who are outside the registry and those excluded from the analyses, limiting the generalizability of our findings. Prior studies have shown that although community-level SES is a very good proxy for individual-level SES, it is not perfect. In a prior study, community-level SES was shown to be independently associated with adverse health outcomes, after adjusting for individual-level SES.^25^ This implies that the combination of neighborhood and individual effects of poverty are synergistic, suggesting that the associations between SES and FS might be underestimated in our study. Despite controlling for medications prescribed, we were unable to account for adherence, RA severity or duration, or changes in SES over time in our study. Furthermore, we were unable to evaluate the interplay between SES, FS, and other important social determinants, such as safety, access to adequate food, stress and trauma, social inclusion and support, and the ability to exercise.^24,25,26^

## Conclusions

In conclusion, we found important disparities in FS by SES in a national cohort of individuals with RA, despite utilization of rheumatology care. We provide a framework for monitoring disparities in RA in rheumatology practices. Future qualitative research is important to further our understanding of factors that affect FS, including factors outside of medical care that can be intervened on.

## Notes

Supplement. **eTable. **Comparison of Patient Demographic Characteristics Across the Area Deprivation Index Quintiles
