# Supplementary material for: Socioeconomic Disparities in Functional Status in a National Sample of Patients With Rheumatoid Arthritis
Source: JAMA Netw Open. 2021 Aug 4;4(8):e2119400. doi: 10.1001/jamanetworkopen.2021.19400 (PMC8339935; doi:10.1001/jamanetworkopen.2021.19400)
Supplement: Supplement. — eTable. Comparison of Patient Demographic Characteristics Across the Area Deprivation Index Quintiles [file jamanetwopen-e2119400-s001.pdf]

Supplemental Online Content

Izadi Z, Li J, Evans M, et al. Socioeconomic disparities in functional status in a national sample of patients with rheumatoid arthritis. *JAMA Netw Open.* 2021;4(8):e2119400. doi:10.1001/jamanetworkopen.2021.19400

eTable. Comparison of Patient Demographic Characteristics Across the Area Deprivation Index Quintiles

This supplemental material has been provided by the authors to give readers additional information about their work.

© 2021 Izadi Z et al. *JAMA Network Open.*

eTable. Comparison of Patient Demographic Characteristics Across the Area Deprivation Index Quintiles						
**Cross-sectional Cohort: Patients with RA and at least 1 FS scores documented during the study period, N=83,965.**						
	**1st ADI Quintile** Highest SES level	**2nd ADI Quintile**	**3rd ADI Quintile**	**4th ADI Quintile**	**5th ADI Quintile** Lowest SES level	**P***
**Age, mean (SD)**	63.87 (14.1)	63.33 (14.0)	63.32 (13.7)	63.18 (13.5)	63.32 (13.2)	<0.05
**Female**	12853 (76.9%)	12779 (77.8%)	12895 (76.6%)	13282 (76.6%)	12840 (77.2%)	<0.05
**Race/ethnicity**						<0.05
Non-Hispanic White	12157 (72.7%)	12133 (73.9%)	12478 (74.1%)	12718 (73.3%)	10551 (63.5%)	
Hispanic	715 (4.3%)	888 (5.4%)	771 (4.6%)	803 (4.6%)	1127 (6.8%)	
African American	775 (4.6%)	966 (5.9%)	1132 (6.7%)	1417 (8.2%)	2610 (15.7%)	
Asian	555 (3.3%)	230 (1.4%)	148 (0.9%)	94 (0.5%)	79 (0.5%)	
Other/Mixed	1161 (6.9%)	979 (6.0%)	981 (5.8%)	939 (5.4%)	936 (5.6%)	
Unknown	1355 (8.1%)	1228 (7.5%)	1335 (7.9%)	1380 (8.0%)	1324 (8.0%)	
**Longitudinal Cohort: Patients with RA and ≥ 2 FS scores documented during the study period at least 12-months apart, N=35,385.**						
	**1st ADI Quintile** Highest SES level	**2nd ADI Quintile**	**3rd ADI Quintile**	**4th ADI Quintile**	**5th ADI Quintile** Lowest SES level	**P***
**Age, mean (SD)**	64.00 (13.5)	63.79 (13.5)	63.60 (13.0)	63.56 (12.9)	63.77 (12.6)	0.29
**Female**	5584 (77.8%)	5650 (78.9%)	5375 (76.4%)	5346 (76.7%)	5439 (77.2%)	<0.05
**Race/ethnicity**						<0.05
Non-Hispanic White	5506 (76.7%)	5433 (75.9%)	5300 (75.4%)	5209 (74.7%)	4672 (66.3%)	
Hispanic	284 (4.0%)	322 (4.5%)	251 (3.6%)	218 (3.1%)	369 (5.2%)	
African American	388 (5.4%)	435 (6.1%)	458 (6.5%)	564 (8.1%)	1031 (14.6%)	
Asian	250 (3.5%)	109 (1.5%)	70 (1.0%)	38 (0.6%)	28 (0.4%)	
Other/Mixed	385 (5.4%)	374 (5.2%)	367 (5.2%)	333 (4.8%)	343 (4.9%)	
Unknown	362 (5.1%)	487 (6.8%)	588 (8.4%)	609 (8.7%)	602 (8.6%)	
*Unadjusted p-values were obtained using Chi-squared test for categorical variables and one-way ANOVA test for continuous variables. **RA:** Rheumatoid arthritis; **FS:** Functional status;						

© 2021 Izadi Z et al. *JAMA Network Open.*
